# Recurrent de novo mutations in neurodevelopmental disorders: properties and clinical implications

**DOI:** 10.1186/s13073-017-0498-x

**Published:** 2017-11-27

**Authors:** Amy B. Wilfert, Arvis Sulovari, Tychele N. Turner, Bradley P. Coe, Evan E. Eichler

**Affiliations:** 1 Department of Genome Sciences, University of Washington School of Medicine, Seattle, WA 98195 USA; 2 Howard Hughes Medical Institute, University of Washington, Seattle, WA 98195 USA

**Keywords:** Autism spectrum disorder, De novo mutations, Developmental disorders, Epilepsy, Intellectual disability, Neurodevelopmental disorders, Noncoding SNVs, Whole-exome sequencing, Whole-genome sequencing

## Abstract

Next-generation sequencing (NGS) is now more accessible to clinicians and researchers. As a result, our understanding of the genetics of neurodevelopmental disorders (NDDs) has rapidly advanced over the past few years. NGS has led to the discovery of new NDD genes with an excess of recurrent de novo mutations (DNMs) when compared to controls. Development of large-scale databases of normal and disease variation has given rise to metrics exploring the relative tolerance of individual genes to human mutation. Genetic etiology and diagnosis rates have improved, which have led to the discovery of new pathways and tissue types relevant to NDDs. In this review, we highlight several key findings based on the discovery of recurrent DNMs ranging from copy number variants to point mutations. We explore biases and patterns of DNM enrichment and the role of mosaicism and secondary mutations in variable expressivity. We discuss the benefit of whole-genome sequencing (WGS) over whole-exome sequencing (WES) to understand more complex, multifactorial cases of NDD and explain how this improved understanding aids diagnosis and management of these disorders. Comprehensive assessment of the DNM landscape across the genome using WGS and other technologies will lead to the development of novel functional and bioinformatics approaches to interpret DNMs and drive new insights into NDD biology.

## Background

Every human inherits approximately half of their genetic information from their mother and half from their father. However, a small number of changes, referred to as de novo mutations (DNMs), are not observed in the genome of either parent. These mutations are either newly formed during gamete formation or occur very early in embryonic development and, thus, are unique to the child when compared to the parent. DNMs can range in size from a single nucleotide change to large (>50 kbp) genomic deletions, duplications, or rearrangements (Table 1). Errors during DNA replication, which are not corrected by proofreading mechanisms, or errors in recombination can lead to DNMs [1]. Some regions are more error prone than others due to genomic context and structure [2–5]. Although DNMs can occur anywhere in the genome, the exome, or protein-coding region of the genome, is often investigated first when studying disease [6–8]. Genes that are preferentially, or recurrently, mutated across individuals with disease have led to the discovery of novel disease genes [5, 6, 9–13]. Furthermore, in some instances the same alteration will arise independently in several people with the same or similar disorders [5, 6, 14].

**Table 1:** Summary of the types of DNMs across the genome

DNM class		Size	Description	Average number of DNMs* per genome
Copy number variant (CNV)		> 50 bp	Genomic deletions or duplications that can span both gene regions and noncoding, regulatory regions	0.05–0.16 [8, 23, 26]
Insertion/deletion (indel)		< 50 bp	Insertions or deletions of a small number of nucleotides that alter the reading frame of a protein are called frameshift mutations and typically result in a truncated peptide	2.6–9 [8, 23, 26, 27]
Single-nucleotide variant (SNV)		1 bp	Single base-pair change in the genome	45–89 [3, 7, 8, 23, 27, 28]
SNV subtype	Likely gene disrupting		Results in a truncated peptide, often referred to as stop-gain, stop-lost, or splice-altering mutations	
	Missense		Changes the amino acid sequence of a peptide but does not lead to peptide truncation	
	Synonymous		Mutations that do not alter peptide sequence or length but may alter regulatory regions or RNA processing	
	Noncoding		Changes that occur outside the protein-coding regions of the genome	
Mosaic SNV		1 bp	Single base-pair changes that occur in only a subset of cells in the human body, sometimes referred to as somatic mutations	0.05–22.2 [23, 27, 29–31]
Mosaic CNV		> 50 bp	Deletions or duplications that only occur in a subset of cells in the human body	5e^−4^–7.7e^−3^ [32, 35]

*De novo estimates for CNVs and indels should be considered as a lower bound because of biases against discovery

Neurodevelopmental disorders (NDDs) are a collection of heterogeneous phenotypes diagnosed during early childhood that persist throughout life and include but are not limited to autism spectrum disorder (ASD), intellectual disability (ID), developmental delay (DD), and epilepsy. Combined, NDDs are thought to affect 2–5% of children [15, 16]. Different phenotypes frequently co-occur in the same patient, thus blurring the lines in the classification of children with disease. Much like their phenotypes, the genetic etiology underlying NDDs is highly heterogeneous with varying degrees of genetic overlap and penetrance, or expressivity, across phenotypes [6, 14]. Current treatment strategies for children with NDDs are typically palliative and focus on managing underlying symptoms, such as aggression, seizures, hyperactivity, or anxiety [17, 18], but there are data to suggest that individuals grouped by common genetic etiology share more clinical features [5, 6, 14]. The discovery of novel genes and previously unrecognized subtypes of both syndromic and non-syndromic NDDs holds promise for more tailored therapeutics.

Genomic technologies, such as microarray and next-generation sequencing (NGS), have enabled a more comprehensive interrogation of the entire genome. Recent reductions in cost and more rapid implementation due to improvements in bioinformatics have led to routine use of these assays for diagnostics and genetic testing, particularly for families with children affected with NDDs [19]. The transition from low-resolution microarray-based technology to high-resolution NGS platforms has dramatically accelerated NDD gene discovery [6–8, 10, 12–14, 20–23] and facilitated the exploration of underexplored variant classes, such as DNMs, which was previously restricted to large copy number variants (CNVs) (Table 1) [24–35]. Moreover, NGS has enabled the curation of both common and rare genetic variation to create new population-based resources that have been paramount for the interpretation of variants and elucidation of key pathways and mechanisms underlying NDDs [36–39].

Here, we review the current state of NDDs in the context of DNMs with an emphasis on the implicated genes and genomic regions. Although NDDs may encompass a wide array of phenotypes that affect the developing brain, such as adult neuropsychiatric conditions, we focus here on disorders with pediatric onset. We consider a range of mutations from large CNVs to single-nucleotide variants (SNVs) and explain how the rapid growth of population genetic resources and technology improvements have increased specificity for disease-gene discovery. We summarize functional networks and pathways consistently identified as enriched for DNMs in NDDs, which includes evidence that implicates different regions and cell types of the developing brain. We conclude with a discussion of how this information could improve diagnostics and guide future therapeutics, with specific emphasis on the value of whole-genome sequencing (WGS) over whole-exome sequencing (WES) in both clinical and basic research.

Table 1 provides a description of DNMs typically observed throughout the genome. The average number of DNMs per genome was estimated using WGS (where possible), WES, or array-based techniques. De novo estimates for CNVs and indels should be considered as a lower bound because of biases against discovery. It has been estimated, for example, that > 65% of all CNVs are missed as a result of routine analysis of Illumina-based WGS data [33, 34]. Relative contributions of DNMs to disease vary widely depending on the disease—although DNMs are particularly relevant to NDDs.

## Copy number variation

A CNV was defined originally as a duplicated or deleted DNA segment of ≥ 1 kbp in length; however, with the advent of NGS technology, the definition has been extended to include differences ≥ 50 bp in length (Table 1). Although there are relatively few copy number differences between any two humans (~ 30,000 events), CNVs contribute to many more base-pair differences than SNVs and have a well-recognized role in both human evolution and disease. Array-based comparative genomic hybridization and single-nucleotide polymorphism (SNP) microarrays were some of the first genome-wide approaches used to identify large de novo CNVs in samples from patients diagnosed with NDDs [25, 26, 40–45]. Microarray-based CNV detection in children with ID compared to unaffected controls led to further refinement of the 17q21.31 microdeletion (Koolen-de Vries syndrome) region to only two genes, namely *MAPT* and *KANSL1* [46]. Next, integration of SNV and CNV data confirmed *KANSL1* as sufficient for causation of Koolen-de Vries syndrome [47]. Similar comparisons with SNV data have begun to distinguish two types of CNVs: those where DNMs in a single gene (i.e., monogenic) are sufficient for disease onset (e.g., *KANSL1* and the 17q21.31 microdeletion [47]), and those where dosage imbalance of multiple genes (i.e., oligogenic) may be required to explain fully the phenotype (e.g., 16p12.1 deletion and secondary CNVs [48]). Gene dosage is the number of copies of a particular gene present in a genome, and dosage imbalance describes a situation where the genome of a cell or organism has more copies of some genes than other genes.

**Fig. 1:**
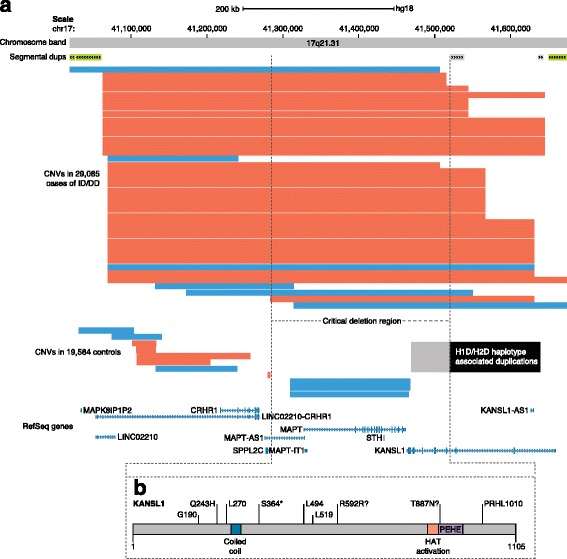
Converging evidence between SNV and CNV data. **a** Very rare atypical deletions define the 17q21.31 minimal region (encompassing *MAPT* and *KANSL1* [46]) using CNVs from 29,085 cases diagnosed with ID/DD and 19,584 controls. *Red* and *blue bars* indicate deletions and duplications, respectively. The *black box* indicates boundaries of H1D (direct haplotype with duplication) and H2D (inverted haplotype duplication) haplotype-associated duplications as determined by genome sequencing. The *light gray box* represents overextended boundaries detected on SNP arrays. **b** Severe de novo SNVs disrupting *KANSL1* were found in patients without the typical microdeletion, which supports *KANSL1* as the gene underlying Koolen-de Vries syndrome [47, 135]. *CNV* copy number variant, *DD* developmental delay, *ID* intellectual disability, *SNV* single-nucleotide variant

Array-based CNV detection is sensitive for large events (CNVs that are at least 25–50 kbp have led to nearly 100% experimental validation when assayed on arrays with 2.7 million probes) [49]. Detection of SNVs and indels by WES has increased specificity and resolution to pinpoint the disease-causing gene or genes disrupted by the candidate CNV (Fig. 1) [25, 26, 49]. Converging independent evidence from microarrays (large CNVs) and WES (likely gene-disrupting (LGD) SNVs), followed by clinical re-evaluation of patients with the same disrupted gene, has led to the discovery of many other disease-causing genes and specific NDD phenotypes, including *CHRNA7* from the 15q13.3 microdeletion region in epilepsy [50, 51]. A recent study suggests that integration of CNV and WES data has begun to converge on specific genes associated with dosage imbalance for 25% of genomic disorders [52]. In other NDD cases, either no single gene has emerged or more than one gene within the critical region has shown evidence of recurrent DNMs, which suggests dosage imbalance of multiple genes might play a role in a specific CNV etiology. Alternatively, the dosage imbalance and disease may be related to the deletion or duplication of noncoding regulatory regions. WGS data will be necessary to explore this largely uncharacterized form of de novo NDD risk [53]. As the amount of WGS data from trios increases to the hundreds of thousands, WGS will likely become the single most powerful tool for discriminating monogenic genomic disorders from those where more than one gene is associated.

### Properties of pathogenic CNVs

Clinically, de novo CNVs are characterized as pathogenic or potentially pathogenic based on size (e.g., ≥ 400 kbp) [46, 54], gene content, de novo status, and overrepresentation in disease cohorts [11, 25, 41, 53, 55, 56]. The number of recurrent de novo CNVs classified as pathogenic ranges from 21 [56] to 41 [14] to 50 [25], depending on diagnostic criteria. The difficulty with CNV diagnosis is that most de novo events rarely re-occur (other than those mediated by known mechanisms [57–59]), which leads to an “n-of-one” problem for the clinician and researcher. Despite the shift to NGS methods, there is a pressing need to consolidate datasets across numerous clinical centers and population control datasets to establish more extensive CNV maps based on hundreds of thousands of patients and controls. Such maps allow clinicians to quickly identify regions of the genome where dosage imbalance is observed in patients but not normal controls. When compared to controls, large inherited CNVs (≥ 500 kbp) are enriched 2.5-fold among cases of NDD [25] and, similarly, de novo CNVs increase ASD risk by twofold [41]. Among NDDs, large de novo CNVs are estimated to account for about 3.7% of cases [8, 11, 60], whereas both inherited and de novo CNVs have been estimated to cause ~ 15% of cases [25, 56].

### Variably expressive vs. syndromic CNVs

**Fig. 2:**
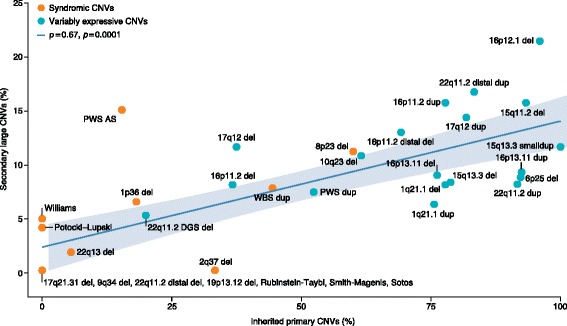
Correlation between the inheritance of variants and incidence of second-site variants. A positive correlation was observed between the proportion of children with developmental delay with inherited primary CNVs (genomic disorders) and children with additional CNVs (Pearson’s product-moment correlation, ρ = 0.67 at significance level of *p* = 0.0001, for disorders affecting ≥ 6 children). Primarily de novo genomic disorders (e.g., Williams-Beuren syndrome) rarely show additional large CNVs, while CNVs (e.g., 16p12.1 deletion) that are primarily inherited have an excess of secondary CNVs compared to population controls (see Girirajan et al. [48] for more detail). *AS* Angelman syndrome, *CNV* copy number variant, *PWS* Prader-Willi syndrome, *WBS* Williams-Beuren syndrome. Adapted with permission from [48]

Classification of recurrent pathogenic CNVs as syndromic or variably expressive depends on the range and reproducibility of phenotypic features observed in patients (Fig. 2) [48]. Recurrent CNVs are syndromic when they are sufficient to result in a highly reproducible set of disease features, whereas variably expressive CNVs result in a broader and more varied spectrum of phenotypic outcomes. As the numbers of clinical reports of patients with the same CNVs increase, it has become clear that a larger fraction of CNVs are variably expressive, with most CNVs manifesting a wide range of clinical phenotypes. For instance, the chromosomal 15q13.3 deletions and duplications are now clearly associated with ID [61], ASD [62], epilepsy [50], and schizophrenia [63] across distinct patient cohorts. Many aspects of these phenotypes have been recapitulated in mouse models [64, 65]. This phenotypic variation and the fact that “unaffected” carrier parents have been identified indicate that these CNVs alone are not always necessary or sufficient to cause disease. Interestingly, variably expressive CNVs are more likely than syndromic CNVs to be inherited and patients with this type of CNV are more likely to carry a secondary large CNV (> 500 kbp) elsewhere in the genome when compared to patients with syndromic CNVs or population controls (Fig. 2). Indeed, patients carrying two or more large inherited and/or de novo CNVs (> 500 kbp) are eightfold more likely to develop an NDD [48]. These observations provided early evidence for an oligogenic CNV model where in addition to the primary recurrent CNV a second rare or de novo CNV or SNV is required at a different locus or gene for a child to develop ID or DD [48, 66–68].

### Parent-of-origin effects

De novo CNVs often arise mechanistically as a result of elevated mutation rates in regions flanked by segmental duplications (long DNA sequences with > 90% sequence similarity that exist in multiple locations across the genome) [69] due to unequal crossing over between the repeats during meiotic recombination [59, 70, 71]. This mechanism causes high rates of DNM recurrence around these duplications, which leads to the identification of syndromic CNVs [46]. There is evidence of a paternal-age effect regarding breakpoint variability due to replication errors in these regions, whereas local recombination biases are mediated by unequal crossing over [72]. For example, over 90% of de novo deletions and duplications associated with the chromosome 16p11.2 microdeletion originate in the maternal germline likely because there is tenfold bias in this region for maternal recombination when compared to male recombination [73]. Indeed, inherited CNVs also show parent-of-origin effect, with a preferential transmission of a CNV to children from one parent over the other (e.g., the transmission of a CNV from mother to child occurs more often than expected by chance). Large, potentially pathogenic CNVs and secondary CNVs show evidence of a significant maternal transmission bias [11, 48, 73, 74] and this observation has been recently extended to private (a rare mutation only found in a single family) loss-of-function SNV mutations in ASD families. Maternally inherited, rare duplications < 100 kbp in size were found to contribute to ASD risk by 2.7%, whereas the equivalent disease attributable fraction for private, inherited LGD SNVs was 7.2% [11]. By comparison, the inherited paternal LGD SNV events contributed a nonsignificant proportion of 1.0% [11]. Although the basis for these transmission biases is unknown, the data are consistent with a “female protective effect” model [11, 74]. This model implies that females carry a higher number of inherited and de novo CNVs than males and so require a greater mutational load for disease onset. Moreover, female carriers of these deleterious events are more likely to transmit them, as they carry a reduced liability, which causes male carriers to be affected disproportionally by these events contributing, in part, to the male bias observed in many NDDs. The observation that ASD females tend to carry more DNMs than males provides further support for this hypothesis [75].

## Protein-coding SNV and indel DNMs

**Fig. 3:**
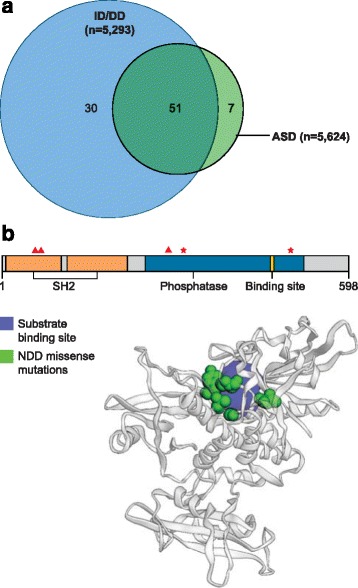
DNM gene overlap and clustered mutations. **a** Venn diagram comparing genes enriched with LGD DNMs in an NDD cohort [39]. There is considerable sharing across two common NDD phenotypes, which suggests considerable shared genetic etiology underlying ASD and ID/DD. The degree of sharing may be indicative of disease severity, where genes that overlap ID/DD and ASD are more likely to be underlying more severe phenotypes and outcomes. **b** PTPN11 shows 3D clustering of missense DNMs in NDD patients (reproduced with permission from [5]). The *top figure* shows the 2D structure of PTPN11 and highlights several key protein domains. The *red triangles* above the 2D structure indicate the location of the amino acid change caused by missense DNMs and the *red stars* indicate residues that have been recurrently mutated in an available NDD cohort. The 3D ribbon structure shows clustering of the missense DNM residues near the protein’s substrate binding site [96]. *ASD* autism spectrum disorder, *DD* developmental delay, *DNM* de novo mutation, *ID* intellectual disability, *LGD* likely gene-disrupting

SNVs (single base-pair changes) and indels (small deletions or insertions < 50 bp in length) are the most common forms of genetic variation in the genome (Table 1) [76]. Patterns of SNVs and indels across the genome have led to many important insights regarding genome evolution, function, and the role of genetic variation in disease [76]. Extensive family-based NGS studies, which include the Deciphering Developmental Disorders (DDD) study, Autism Sequencing Consortium (ASC), and Simons Simplex Collection (SSC), have firmly established the importance of germline DNMs in NDDs [6, 10, 11, 13, 42, 77]. These studies have largely focused on the exome, the most functionally well-characterized portion of the genome. Cumulatively, these and similar studies have identified hundreds of candidate genes involved in at least one NDD phenotype, which highlights both the locus heterogeneity and the shared genetic etiology that underlies these disorders [6, 78] (Fig. 3). Protein-coding DNMs can be grouped into three classes based on functional impact: 1) LGD (stop codon, frameshift, splice donor, and acceptor), 2) missense, and 3) synonymous mutations. Although the overall rate of DNM, in general, does not differ between affected and unaffected siblings, patients with NDDs show an enrichment for LGD and missense DNMs [8, 10, 12, 13, 79]. Moreover, synonymous mutations that play a role in regulating gene expression have been implicated in both NDDs and neuropsychiatric disorders more broadly [6, 10, 53, 80].

### LGD mutations

LGD or protein-truncating variants are the best-characterized class of DNMs because of their straightforward mechanism of action and abundance in children with NDD. For example, there was a twofold excess of LGD DNMs in ASD patients versus their unaffected siblings [13, 79]. LGD DNMs are estimated to contribute to 6–9% of all NDD diagnoses, with the variability in estimates attributed to differences in diagnosis, DNM criteria, and study design [6, 8, 10, 11]. A clear burden of LGD DNMs can be detected within a heterogeneous cohort of NDD individuals, and recurrence has been used to identify specific genes that contribute to the disease [6, 9, 10, 12, 13, 81–85]. Recent availability of population-level genetic data from tens of thousands of individuals has led to improved gene-specific mutation rate estimates, which enables the identification of genes enriched for various classes of exonic DNMs in NDDs [9, 12]. These same data have also been used to improve interpretation of benign and pathogenic LGD DNMs; however, strict filtering against population controls should be used with caution as it may lead to false negatives [6, 9, 12, 36, 81].

Curation of a DNM database of NDD and other disease studies has facilitated the identification of genes [39]. We find that 58% (51/88) of genes with recurrent mutations in NDD patients have at least one individual with ID/DD and one individual with ASD listed as their primary phenotype (Fig. 3a). For example, the database identified only seven genes specific to ASD: *SPAST*, *S100G*, *MLANA*, *LSM3*, *HMGN2*, *WDFY3*, and *SCN1A. SPAST* is a common causal gene of autosomal dominant hereditary spastic paraplegia, a phenotype that is very distinct from the characteristic traits of individuals with ASD [86]. Several studies have found that individuals with DNMs in the same gene are more phenotypically similar despite the initial ascertainment criteria for the study [5, 6, 14, 82–84, 87, 88].

Although there are overlapping genes between ASD and ID/DD phenotypes, gene sharing does not necessarily result in identical phenotypes across patients. For example, the DDD reported that 56% of their cohort carried an LGD or missense DNM in a known epilepsy gene even though only a quarter of these individuals had reported epilepsy or seizure phenotypes [6]. DNMs in such genes may be modifying the severity of the primary phenotype. Indeed, the presence of DNMs in known ID genes has been associated with a more severe phenotype in patients with ASD and some neuropsychiatric disorders, such as schizophrenia, which supports this idea [10, 89]. Although similar phenotypes are more likely to have a shared genetic etiology, a common genetic etiology does not always indicate the same phenotype, which highlights the importance of balancing detailed phenotype–genotype correlations with sample size to optimize power for gene discovery [6]. Consideration of the criteria used to establish a diagnosis is also important because changes in guidelines could result in misleading genetic sharing across NDDs. As diagnostic guidelines are changed patients enrolled in studies should be re-evaluated using the new criteria and both the clinical and molecular phenotypes should be considered when drawing conclusions.

Some recurrent mutations in specific genes (Table 2), however, show preferential primary diagnoses. For example, LGD mutations in *GATAD2B* have been observed exclusively in ID/DD cases whereas LGD mutations in *CHD8* have been biased toward ASD cases, which means that some cases reported as ID/DD also carry an ASD diagnosis (Table 2). *GATAD2B* plays a key role in cognition and synapse development and has been previously implicated in ID pathogenesis [90]. *CHD8* codes for a DNA-binding protein involved with chromatin modification, which when knocked down causes decreased expression of genes involved in synapse function and axon guidance as well as macrocephaly in zebrafish and similar features in the mouse [91, 92].

**Table 2:** Top 26 LGD de novo-enriched genes associated with NDDs

Gene	NDD (n = 11,505)	ID/DD (n = 5303)	ASD (n = 5624)	Epilepsy (n = 532)	Weighted ASD:ID/DD ratio	SFARI gene score	SFARI report count
*ARID1B*	45	36	9	0	0.236	1	23
*ANKRD11*	41	35	4	2	0.108	2	27
*KMT2A*	36	29	5	2	0.163	2	11
*ADNP*	26	20	6	0	0.283	1	18
*DDX3X*	24	22	2	0	0.086	3	4
*SYNGAP1*	24	17	6	1	0.333	1	34
*ASXL3*	22	19	3	0	0.149	1	8
*DYRK1A*	20	15	5	0	0.314	1	28
*SCN2A*	19	10	9	0	0.849	1	40
*SETD5*	18	17	1	0	0.056	1	15
*CTNNB1*	17	16	1	0	0.059	3	16
*POGZ*	17	13	4	0	0.290	1	20
*MED13L*	16	14	2	0	0.135	2	13
*CHD8*	15	4	11	0	2.593	1	22
*CHD2*	14	8	4	2	0.472	2	18
*EP300*	14	13	1	0	0.073	4	13
*KAT6B*	14	13	0	1	0.000	N/A	N/A
*MECP2*	13	7	4	2	0.539	2	58
*AHDC1*	12	11	1	0	0.086	3	6
*FOXP1*	11	9	2	0	0.210	2	24
*TCF4*	11	10	1	0	0.094	S	28
*WDR45*	10	7	0	3	0.000	N/A	N/A
*GATAD2B*	10	10	0	0	0.000	N/A	N/A
*KAT6A*	10	9	1	0	0.105	3	7
*SHANK3*	10	4	6	0	1.414	1	56
*TCF20*	10	9	1	0	0.105	3	5

Table 2 lists 26 genes with the most LGD DNMs across 11,505 NDD cases [39]. The genes listed show considerable sharing and specificity of genetic drivers across three common NDD phenotypes (ASD, ID/DD, and epilepsy), which is highlighted by the weighted ASD:ID/DD ratio calculated by comparing the frequency of DNMs per gene for each disorder. The Simons Foundation Autism Research Initiative (SFARI) gene score and report count demonstrate the variability in our understanding of the top contributing DNM genes and highlight several genes not currently included in the SFARI database [93].

### Missense mutations

Missense mutations are single base-pair changes that occur within the genic regions of the genome and alter the amino acid specified by a codon. Although the impact of missense DNMs on gene function is not as easy to interpret, studies have identified a modest but statistically significant excess of recurrent DNMs in NDD cohorts when compared to population controls [5, 6, 10, 85]. In fact, population controls have been crucial to predicting the functional impact of missense DNMs [9]. When restricting to genes that are more intolerant to mutation or DNMs that are more severe, the signal from missense DNMs becomes stronger [5, 81]. Genes with a significant excess of recurrent missense DNMs have been identified [5, 6, 9, 12, 85] and, interestingly, not all genes that show enrichment for missense DNMs are enriched for LGD DNMs [85]. Furthermore, the phenotype observed across individuals with DNMs in the same gene can differ if the DNM is missense or LGD [6]. For example, the DDD study reported marked differences between missense and LGD mutations in the Cornelia de Lange syndrome gene *SMC1A*, noting that individuals with LGD DNMs lack the characteristic facial dysmorphia observed in individuals with missense Cornelia de Lange syndrome-causing DNMs [6]. Similarly, DNMs in *SCN2A*, which encodes a sodium ion channel protein, are reported nearly as frequently in ASD as in ID/DD cases (Table 2), with the resulting phenotype determined by DNM function [94]. Loss-of-function DNMs in this gene associate with ASD whereas gain-of-function DNMs lead to infantile epilepsy and ID [94].

Several recent studies have shown that missense DNMs are more likely to cluster within protein-functional domains that aggregate in both the two- and three-dimensional structure of the protein (Fig. 3b) [5, 14, 95, 96]. An extreme example of such clustering is recurrent site mutations. Predictably, these clustered DNMs often define important ligand–receptor, transcription factor binding, or transmembrane domains important to the function of the protein [5, 6, 14]. For example, a recent study of individuals with ASD and ASD-related disorders identified a cluster of missense DNMs in the GEF1 domain of *TRIO*, a gene involved in the Trio-Rac1 pathway [97]. Functional studies of these DNMs confirmed that they disrupted normal *TRIO* function and significantly altered dendritic spine density and synapse function, which demonstrates how these findings can be used to elucidate pathways and begin to propose therapeutic targets [97]. Other approaches for assessing the functional impact of missense DNMs include computational predictions of pathogenicity to generate short lists of the most likely candidate variants, or high-throughput functional assays to confirm or refute the impact of an amino acid change on gene function [98, 99].

### Mosaic mutations

Mosaic mutations occur as a result of postzygotic mutation, which leads to a subset of cells that differ genetically from the other cells in the body. These mutations, also referred to as somatic mutations, are an important but particularly problematic source of mutations that are frequently either missed or reported incorrectly as a DNM [100]. Specifically, mutations that occur in only a subset of the parent’s cells can lead to false positive DNM calls in patients or false negative calls if the DNM does not occur in a sufficient number of the patient’s cells [100]. In addition to germline DNMs, mosaicism has been explored within the patient as another class of DNM that might contribute to NDDs. Improvements in variant callers (computational algorithms that identify genetic differences in an individual relative to a genetic reference panel), and deep- and multi-tissue sequencing, have facilitated the detection of mosaic DNMs and identified a role for mosaic DNMs in NDDs [29–31, 100, 101]. Notably, estimates of early embryonic mutation rates (e.g., mutations that occur postzygotically) are expected to be comparable or slightly higher than germline mutation rates and show a similar mutational spectrum [102]. Several studies have estimated a wide range of postzygotic mutation frequencies (1–7.5%) depending on whether the whole genome or only the exome is considered and the depth at which the samples were sequenced (deep sequencing offers more power to detect low-frequency mosaic mutations) [23, 29–31, 100, 101]. These studies also detected an increased burden of mosaic DNMs in the coding regions of the genome among NDD patients and report that 3–5% of NDD cases are likely attributable to mosaic DNMs. Mosaic mutations in the parents could explain cases of recurrence in families with otherwise de novo causes of NDD [29–31, 100, 103]. Mosaic mutations might also help explain some of the variable expressivity or incomplete penetrance observed in NDDs, depending on the degree to which the targeted organ is affected [103].

## Noncoding SNVs and indels

Noncoding DNMs have been explored only recently because of the higher cost of WGS, which limits our understanding of the functional importance of nongenic mutation (Table 1) [7, 53]. A small ASD study (53 families) reported an enrichment of noncoding DNMs near ASD-associated genes but concluded that larger sample sizes would be needed [7, 53]. Several studies submitted or recently published have substantially increased sample sizes and used WGS to interrogate various classes of DNM across the genome [8, 104–106]. Most of these studies show evidence of DNM enrichment in putative regulatory DNA and one study suggests that such mutations may explain an additional 3–5% of NDD cases, although these estimates represent, almost certainly, a lower bound [8]. Two studies considered 516 families and focused only on a small fraction of the noncoding genomes thought to be the most functionally relevant (3′ and 5′ untranslated regions, known enhancers, and evolutionarily conserved elements) [8, 104, 105]. These preliminary findings are intriguing because they suggest that noncoding DNMs may be one of the major contributors of disease risk. Furthermore, the results provide evidence that multiple DNMs at different locations occur more frequently in the genomes of ASD patients compared to their unaffected siblings [8, 104, 105]. These multiple events are especially enriched in noncoding or protein-coding regions for genes previously implicated in ASD, which provides additional support for an oligogenic model of NDD, in this case, associated exclusively with DNM [8].

### Parent-of-origin effects

**Fig. 4:**
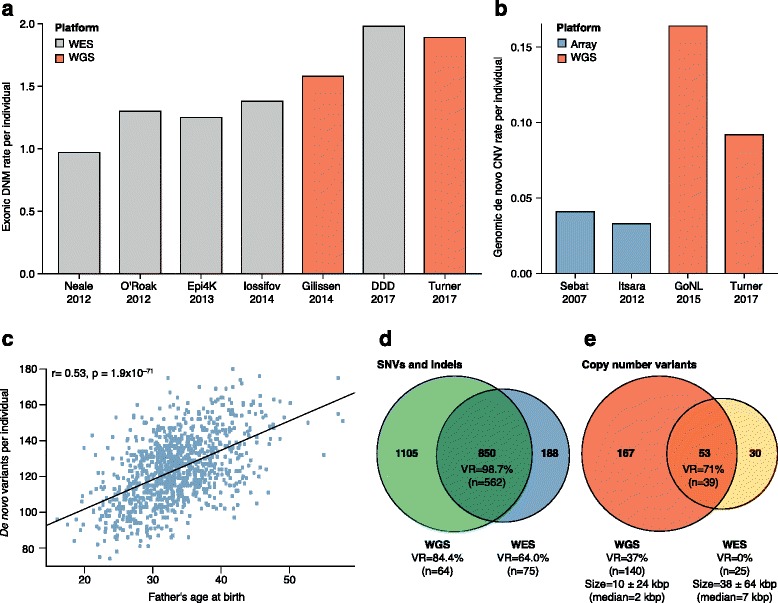
Platform comparisons for DNM detection. **a** Rate of exonic DNMs reported across six WGS and WES studies [6–8, 10, 136, 137]. The transition to WGS has generally led to marked improvements in estimates of the average number of DNMs per exome, although improved methodology has also facilitated better DNM estimates for WES. Although the 2017 DDD study used improved DNM calling estimates, they also applied more permissive calling criteria for DNMs than the other WES studies to improve sensitivity. For example, 15% of individuals in the DDD study carry four or more DNMs, accounting for 31% of the DNMs reported in the study, with some individuals carrying as many as 36 DNMs per exome. **b** Rate of genomic CNVs reported across four SNP microarray and WGS studies [8, 24, 26, 138]. WGS resulted in a noticeable increase in the average number of de novo CNVs per genome due to the improved resolution to detect smaller (< 1 kbp) CNVs. **c** Relationship between the number of DNMs per child and father’s age at birth (*blue dots*) for 986 individuals from a recent study of autism (reproduced with permission from [8]). The estimated rate of increase in DNMs per year of paternal age (*black line*) is 1.64 (95% CI 1.48–1.81) [8]. **d** Venn diagram comparing DNM yield for WGS and WES from a recent study of 516 autism families (reproduced with permission from [8]). Validation rates (*VR*) and number of DNMs tested are listed for WGS only, WES only, or both. DNMs discovered by WGS only or both have higher VRs than WES-only DNMs, likely due to more uniform coverage of the exome by WGS. **e** Venn diagram comparing yield for de novo CNVs between WGS and WES from a recent study of 53 ASD families (reproduced with permission from [53]). Average CNV size was 10 ± 24 kbp (WGS) and 38 ± 64 kbp (WES) and median was 2 kbp (WGS) and 7 kbp (WES). De novo CNVs discovered by both WGS and WES had higher VRs than for de novo CNVs discovered by WGS. None of the de novo CNVs discovered by WES alone were validated. *CNV* copy number variant, *DD* developmental delay, *DDD* deciphering developmental disorders, *DNM* de novo mutation, *SNP* single-nucleotide polymorphism, *VR* validation rate, *WES* whole-exome sequencing, *WGS* whole-genome sequencing

The number of DNMs in a child increases with advancing paternal age at conception [6, 8, 10, 12, 28, 107], which is thought to be due to more cell divisions required to produce the germ cells in males [107]. Recent WGS studies estimate that fathers contribute an extra 1.32–1.65 DNMs per year of age (Fig. 4c) [8, 28]. There have also been reports of an increase in DNMs due to maternal age, although the effect is modest compared to the paternal contribution [3, 6, 10, 28]. A recent WGS study of 1548 control trios reported an increase of 0.32–0.43 DNMs per year of maternal age, and a WES study of approximately 4000 NDD trios reported an increase of 0.32–1.40 DNMs per year of mother’s age [6]. Despite the lower overall contribution of DNMs per year of maternal age, the recent WGS study found that some regions of the genome are more likely to mutate in either mothers or fathers [28]. Although the basis for this sex-specific regional bias is not known, the bias could have profound effects on our understanding of disease risk by DNM, especially the parent-of-origin and female protective effects that have been observed in certain NDDs.

### WGS vs. WES of patient genomes

Microarray data provided some of our first glimpses into the importance of DNM with respect to NDD, and WES further refined the model—helping to understand the contribution of specific genes and different variant classes. The recent drop in WGS costs has led to a shift from WES-based studies to WGS [7, 8, 108]. However, the price differential between WGS and WES is still a significant consideration, which limits the number of samples studied and, therefore, power for gene discovery. With respect to the clinic, WGS will ultimately replace WES as the primary method for diagnosis and disease gene discovery for three reasons.

The first reason is increased diagnostic yield. Direct comparisons of WES and WGS have found that WGS provides more uniform coverage over protein-coding regions when restricting to regions covered by both platforms [7, 8, 53, 109]. For example, in gnomAD 89.4% of the exome was covered by WES with at least 20× coverage while 97.1% was covered by WGS at this coverage threshold [36]. It should be noted that the WES data in these comparisons are typically generated before the WGS results and that the age of the WES platform may account for some of these differences [7, 8, 53]. More uniform coverage allows for improved DNM detection and discovery of protein-affecting DNMs that would otherwise be missed (Fig. 4d) [7, 8, 53]. In fact, there has been a trend of increasing DNM rates for SNVs as the field transitions from WES to WGS; some of this gain can be attributed to improvement in the methodology used in WES studies and the rest is due to better coverage and data quality (Fig. 4a) [109].

Second, CNV detection with capture-based methods is severely limited and many CNVs that affect genes are missed [7, 8, 53]. WGS provides the greatest sensitivity for the detection of CNVs (Fig. 4b, e). There is now evidence that smaller gene-disruptive CNVs (below the level of standard microarray analyses and missed by WES) are twofold enriched in cases of ASD when compared to unaffected siblings [8]. Similarly, a recent WGS study of individuals with ID who were microarray and WES negative for a diagnostic variant found that 10% of their cases carried a structural variant missed by the other two platforms [7]. A similar case has been made for indels where high-quality events are much more readily identified in WGS when compared to WES (Fig. 4d) [110].

Third, WGS provides access to the functional noncoding portions of the human genome. Access to both the coding and noncoding regions of the genome simultaneously may be particularly relevant if the oligogenic model holds [111]. A recent study, for example, estimated that individuals with three or more DNMs of interest account for about 7.3% of simplex ASD [8], although such multiplicities may be expected if we are enriching for pathogenic mutations. Ultimately, WGS provides a more accurate and more complete picture of the genetic etiology underlying NDDs and the genetic risks that contribute to disease in individual patients (Fig. 4d, e).

## Functional gene networks and tissue enrichments

Biological functions of the genes affected by DNM show distinct and interconnected pathways. In the case of ASD, for example, three pathways appear to be important. First, chromatin remodeling is frequently highlighted [77, 85, 112–114]. Chromatin remodeling appears to function particularly early in development, as early as 7 weeks post-conception, and is associated with transcriptional regulation, chromatin modification [115], and nucleosome remodeling factors [116]. Second, pathways associated with cell proliferation and neuronal migration are expressed later in development and contribute to potential overgrowth or undergrowth of neuronal phenotypes through signaling from the MET receptor tyrosine kinase [117]. A recent study characterized molecular effects of LGD DNMs in the gene *EBF3* and reported that GABAergic neuronal migration and projections were abnormal [118]. Third, synaptic networks and long-term potentiation pathways are frequently highlighted and these genes reach their highest levels of expression postnatally [112]. Such genes have been reported as differentially expressed, for example, in the postmortem brains of patients with ASD [119, 120]. Exome sequencing studies of ASD and ID have identified genes important in the function of postsynaptic neurons, such as calcium signaling and long-term potentiation [77, 112]. *CACNA1D*, for example, encodes the calcium channel protein Cav1.3 and has been found to become hyperactive due to gain-of-function DNMs in ASD [121].

**Fig. 5:**
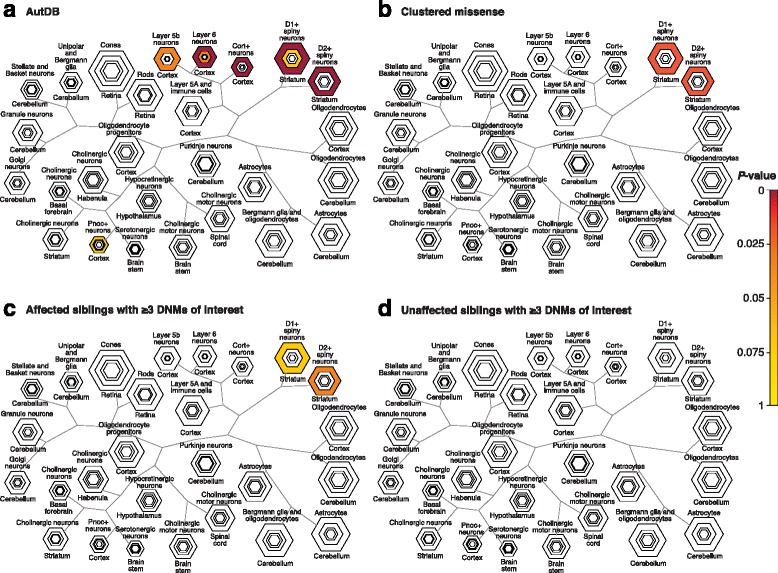
Different lines of evidence support cell-specific enrichment for striatum. **a** A curated list of 899 genes from the Autism Database (AutDB) shows cell-type enrichment in the cortex (layer 6, Benjamini-Hochberg adjusted enrichment *p* = 2 × 10^−5^ at specificity index probability (pSI) of 0.05) and striatum (for D1+ and D2+ spiny neurons, adjusted *p* = 8 × 10^−6^ and *p* = 8 × 10^−4^ at pSI = 0.05) tissues. **b** Enrichment results using 211 genes with rare (frequency < 0.1%) clustered missense mutations [5] (for both D1+ and D2+ spiny neurons, adjusted *p* = 0.005 at pSI = 0.05). **c** NDD patients with ≥ 3 DNMs (for D1+ and D2+ spiny neurons, adjusted *p* = 0.08 and *p* = 0.01 at pSI = 0.05) (reproduced with permission from [8]). **d** Unaffected siblings with ≥ 3 DNMs show no cell-type specific enrichment [8] (for D1+ and D2+ spiny neurons, adjusted *p* = 0.84 and *p* = 0.90 at pSI = 0.05) (reproduced with permission from [8]). Candidate cell types were identified using the Cell-type Specific Enrichment Analyses tool [37]. The resulting honeycomb images show increasingly stringent pSI thresholds in each nested hexagon, where *darker colors* denote *p* values of higher significance. *DNM* de novo mutation

In addition to functional protein–protein interaction and co-expression networks, there have been attempts to identify specific tissues and cell types enriched for genes with DNM. Consistent with previous reports [38], both cortical [122] and striatum neurons (spiny D1+ and D2+) [38, 123] are significantly enriched in ASD risk genes. Co-expression networks of candidate ASD genes identified mid-fetal layer 5/6 cortical neurons as a likely point of convergence for these genes [122]. Four independent analyses of DNMs in NDD cohorts have also recently converged on the same striatum medium spiny neurons (D1+ and D2+). These include known ASD genes from SFARI (AutDB) [94], genes with clustered de novo or very rare missense mutations [5], genes in affected individuals with ≥ 3 DNMs of interest [8] (Fig. 5), and more recently, genes from known pathogenic CNV regions that also show an enrichment for de novo SNVs [52]. Notably, striatal circuits have been postulated to account for ASD-specific repetitive motor behavior [124]. Strong support for this model comes from both MRI studies of ASD children [125] and rodent genetic models of ASD, including knockout models of *Fmr1*, *Shank3*, *Cntnap2*, *Cntnap4*, 16p11.2 heterozygote models, and *Met* receptor knockouts—all of which lead to abnormal striatal structure and function in rodents [124]. Thus, the striatum represents an opportunity for exploring the etiology of behavioral and motor deficits in a specific subset of ASD patients and other NDDs with shared dysfunctions.

## Implications of DNMs across NDDs

In aggregate, de novo protein-coding SNVs, indels, and CNVs account for 13–60% diagnostic yield for NDD cases depending on the disease or diagnostic criteria [6, 7, 10, 14, 21, 53]. For example, protein-coding DNM SNVs in ASD have an estimated attributable fraction of ~ 15% of cases [8], with de novo CNVs accounting for an additional 2.9–6% [8, 10, 11]. Because noncoding mutations are understudied and difficult to interpret, diagnostic yield is currently low and generally reported on a case-by-case basis. However, about 2–4% is a lower bound across NDDs [8]. CNVs and LGD DNMs tend to underlie more severe phenotypes, whereas missense DNMs have been implicated in less severe forms of disease, such as high-functioning ASD [6]. The clustering of missense DNMs in the 2D or 3D protein structure is likely to provide important insights into function and specific targets for future discovery and therapeutics.

WGS has facilitated a more comprehensive assessment of DNM and early reports suggest a modest signal in a subset of noncoding regions relevant to fetal brain development [8, 53, 104]. Moreover, both CNVs and DNM SNVs provide support for the potential role of multiple de novo and private mutations in disease manifestation and severity of disease. The oligogenic model (few de novo or private mutations of large effect) requires a shift from WES to more comprehensive WGS analysis of families, as some of the contributing mutations may be located in the noncoding regions of the genome. If the genetic odyssey for patients ends at the discovery of a likely pathogenic event identified by microarray or exome sequencing, other mutations contributing to disease severity could be overlooked in the absence of WGS data. We believe it imperative that every family with a child with an NDD be considered for WGS so that all pathogenic mutations are discovered, which will lead to improved diagnostic prediction and potential therapeutic intervention. This should become increasingly feasible as sequencing costs continue to drop [19] and WGS becomes one of the most inexpensive diagnostic tests offering the most information.

The role of inherited mutations is also very important. Interactions between DNMs and common variants have been relatively underexplored, but one study reported that, unlike DNMs, which tend to act more akin to a single variant of large effect, common variants act in an additive manner, distinct from DNMs [126, 127]. The polygenic model assumes a large number of disease-causing mutations, each with small effect size and low penetrance, which, when combined with environmental factors, cumulatively suffice to cause disease [128]. More recently, the omnigenic model was introduced, which assumes that through regulatory networks all genes expressed in the disease tissue of interest will affect other genes, making all genes relevant to disease; this model was supported in the context of several highly polygenic traits: human height, autoimmune disorders, and neuropsychiatric disorders, such as schizophrenia [129]. These models are not mutually exclusive because supporting evidence exists for all three in the literature; however, they are likely to identify different subtypes of NDD.

Although the current list of gene targets is still incomplete, the known genes that are enriched with DNMs provide a foundation not only for developing molecular therapies for NDDs [68] but also for grouping patients and developing genotype-first diagnostic approaches appropriate for each group [130]. The latter can lead to clinically actionable opportunities for NDD patients. For instance, an ASD patient that harbors a 22q11.2 deletion may need to be under surveillance for cardiovascular and calcium metabolism problems, and signs of psychotic disorders [131]. Similarly, the inheritance model of deleterious CNVs may inform treatment options; for instance, paternally inherited 15q11-q13 deletions, the locus underlying the imprinting disorder Prader-Willi syndrome, may require psychiatric and endocrine system screening [131].

## Conclusions

Moving forward, WGS of patients and their families will provide increased sensitivity for disease-variant detection. Determining the relative contribution of monogenic, oligogenic, or polygenic models to NDDs will require such datasets. In this regard, a major challenge will be to establish the functional relevance of noncoding portions of the genome before WGS findings can reach the clinic. This will require the development of large-scale functional assays and establishing pathogenicity criteria. More importantly, despite the benefits of WGS, there are still limitations. The most popular WGS methods fragment the genome into ~ 400-bp inserts generating pairs of short (~ 150 bp) sequence reads. Not all regions or types of genetic variation can be readily assayed using this platform alone [34, 132, 133] and the most recent studies have suggested that > 65% of human structural variants (< 2 kbp in size) are being missed [33, 34, 133]. Deep WGS and comprehensive variant detection are not equivalent. Complete resolution of genetic variation in a human genome, we believe, requires the de novo assembly of genomes as opposed to simply aligning short reads to a reference sequence [134]. Long-read sequencing technologies (such as Oxford Nanopore and Pacific Biosciences) have brought us closer to achieving this goal; however, further advances in throughput and analytic approaches will be required to resolve more complex structural variants, such as expansions of large tandem repeats [134] or variation in duplicated regions of our genome. Although the mutations and the genes underlying many NDDs have been discovered, those that remain undiscovered will require a more complete assessment of the genome to understand fully the biology underlying the disorders.

## Abbreviations

ASC: Autism Sequencing Consortium
ASD: Autism spectrum disorder
CNV: Copy number variant
DD: Developmental delay
DDD: Deciphering developmental disorders
DNM: De novo mutation
ID: Intellectual disability
LGD: Likely gene-disrupting
NGS: Next-generation sequencing
SFARI: Simons Foundation Autism Research Initiative
SNP: Single-nucleotide polymorphism
SNV: Single-nucleotide variant
SSC: Simons Simplex Collection
VR: Validation rate
WES: Whole-exome sequencing
WGS: Whole-genome sequencing
